# Sample Tracking Tool: A Comprehensive Approach Based on OpenArray Technology and R Scripting for Genomic Sample Monitoring

**DOI:** 10.3390/diagnostics15020149

**Published:** 2025-01-10

**Authors:** Giulia Trastulli, Giulia Calvino, Bruno Papasergi, Domenica Megalizzi, Cristina Peconi, Stefania Zampatti, Claudia Strafella, Carlo Caltagirone, Emiliano Giardina, Raffaella Cascella

**Affiliations:** 1 Genomic Medicine Laboratory UILDM, IRCCS Santa Lucia Foundation, 00179 Rome, Italy; 2 Department of Systems Medicine, Tor Vergata University, 00133 Rome, Italy; 3 Department of Science, Roma Tre University, 00146 Rome, Italy; 4 Department of Biomedicine and Prevention, Tor Vergata University, 00133 Rome, Italy; 5 Department of Clinical and Behavioral Neurology, IRCCS Santa Lucia Foundation, 00179 Rome, Italy; 6 Department of Chemical-Toxicological and Pharmacological Evaluation of Drugs, Catholic University Our Lady of Good Counsel, 1010 Tirana, Albania

**Keywords:** sample tracking, OpenArray^TM^ technology, Random Match Probability, R for genetic data, R programming

## Abstract

**Background/Objectives**: Centralizing genetic sequencing in specialized facilities is pivotal for reducing the costs associated with diagnostic testing. These centers must be able to verify data quality and ensure sample integrity. This study aims at developing a protocol for tracking NGS-analyzed samples to prevent errors and mix-ups, ensuring proper quality control, accuracy, and reliability in genetic testing procedures. To this purpose, a protocol based on the genotyping of a panel of 60 single-nucleotide polymorphisms (SNPs) by OpenArray^TM^ technology was employed. **Methods**: The protocol was initially tested on a cohort of 758 samples and subsequently validated on a cohort of 100 samples. Furthermore, its ability to accurately detect identical and different samples was evaluated through a simulation test conducted on an additional 100 samples. **Results**: In total, 55 probes achieved a call rate ≥90% and were subjected to the sample matching process performed by an R tool specifically developed. The SNP panel achieved a random match probability of 3.29 × 10^−15^, proving its suitability for efficiently tracking samples and rapidly identifying any errors or mix-up during the analytical processing. **Conclusions**: The features of OpenArray^TM^ technology, cost-effectiveness, rapid analysis, and high discriminative power make it a suitable tool for sample tracking. In conclusion, this method represents a valuable example for promoting laboratory centralization and minimizing the risks related to different laboratory procedures and the management of a high number of samples.

## 1. Introduction

The advancements in high-throughput technologies have led to significant progress in medicine and healthcare by generating huge amounts of data in the field of genomics and other omics sciences [1]. In this context, the harmonization and standardization of such volumes of data are crucial to allow their management, integration, and application into medical research and practice along with the implementation of robust bioinformatic approaches. Applying bioinformatics pipelines became extremely important with the introduction of next-generation sequencing (NGS) technologies into medical practice [2]. In particular, NGS allows the identification of DNA variations associated with different genetic disorders in a time-efficient and cost-effective manner [3]. In the era of precision medicine, introducing genetic data into healthcare services proved to be a powerful tool to facilitate disease diagnosis, treatment, and prevention [4]. In this scenario, the implementation of NGS techniques (whole-exome sequencing (WES) and whole-genome sequencing (WGS)) in Italy led the Ministry of Health to publish a national plan for omics sciences, aimed at (i) transferring genomic knowledge into healthcare practice; (ii) increasing the effectiveness of disease prevention, diagnosis, and treatment, taking into consideration individual differences in terms of genetic features, lifestyle, and environment; and (iii) fostering cultural, scientific, and technological innovation in the healthcare system [5]. From this perspective, centralizing genomic analysis in specialized centers is advantageous for standardizing lab protocols, facilitating the simultaneous processing of numerous samples, and maximizing the efficiency of staff, reagents, and laboratory equipment use. However, establishing centralized centers for data production and interpretation requires the development of specific procedures to ensure the traceability of samples, their identity, and the quality control of genomic data. This verification process is essential for guaranteeing the accuracy of diagnostic outcomes. In large-scale investigations or everyday diagnostic operations, the ability to accurately track and identify specimens strengthens the reliability of the collected data. This study aims to develop a robust framework for systematically tracking and assessing samples, thereby enhancing the integrity and validity of NGS-derived data. In a context where samples may originate from different biological sources and medical centers, implementing such a protocol into laboratory activities can effectively enhance accuracy and minimize errors and mix-ups during sample processing. In particular, a molecular assay using OpenArray™ (OA) ( ThermoFisher Scientific, Waltham, MA, USA) technology was designed and developed to determine the identity of biological samples tested using WES. As shown in previous works, the OA approach is a rapid and cost-effective way to simultaneously screen many samples for single-nucleotide polymorphisms (SNPs) employed for different purposes [6,7,8,9]. The comparison between OA and WES data was performed using a tool developed by R software (v. 4.2.3) to simplify the sample identification and matching process. The protocol was initially tested on a cohort of 758 samples, and subsequently, it was validated on a cohort of 100 samples. Moreover, the method’s performance in detecting samples from different patients was also evaluated by a simulation test conducted on 100 additional samples.

As laboratory activities become increasingly centralized, the development of the OA panel and R tool represents a device that can meet the need for sample traceability and control and improve the robustness and reliability of genetic data. In addition, this method also reduces costs through economies of scale, making molecular diagnostics more sustainable and accessible.

## 2. Materials and Methods

### 2.1. Cohort Description

The study was performed on a total of 958 DNA samples derived from patients who accessed the Genomic Medicine Laboratory-UILDM at the Santa Lucia Foundation IRCCS for diagnostic purposes from 2021 to 2023. All patients were subjected to WES (through Illumina Next Seq 550 system, Illumina, San Diego, CA, USA) to confirm clinical diagnosis of various genetic disorders, including retinal inherited disorders, autism spectrum disorders, neuromuscular conditions (limb-girdle muscular dystrophies, facio-scapulohumeral dystrophy), and polycystic kidney disease.

The samples were divided into three sub-cohorts. Specifically, a group of 758 patients was utilized to test the developed protocol, while 100 patients served as the validation group and 100 subjects were employed for the simulation test. Each patient provided informed consent and was assigned a unique alphanumeric and progressive code. Although the selection of subjects was independent of age and biological sex, the study cohort included a female–male (F/M) ratio of 44:56 and an average age of 45.6 ± 21.5 years.

This work was supported by the Ethics Committee of Santa Lucia Foundation IRCCS (CE/PROG.650 approved on 1 March 2018). Written informed consent for participation in this study was provided by the participants.

### 2.2. Selection of SNPs

**Figure 1:**
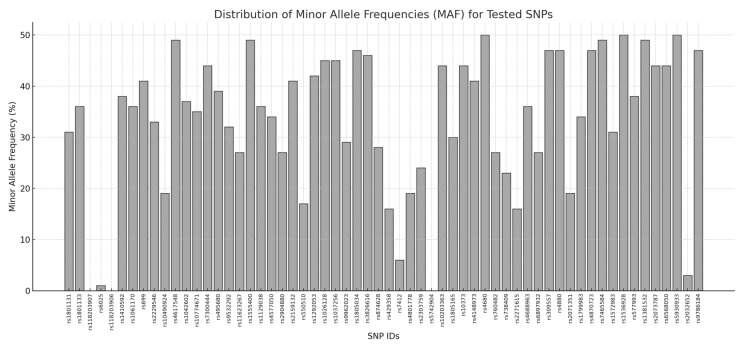
Distribution of minor allele frequencies (MAFs) for all tested SNPs. The x-axis displays the SNP IDs, while the y-axis represents the MAF values expressed as percentages. Each bar corresponds to the MAF of a specific SNP.

A customized panel of 60 SNPs was designed, taking into account specific criteria for SNP selection: (i) a minor allele frequency (MAF) > 1% in the European (EUR) general population; (ii) localization within genomic regions covered by the WES assay; (iii) an average coverage > 20X on the WES platform. These criteria allowed the selection of 55 SNPs of interest, whereas the remaining 5 variants were chosen with a lower MAF (<1%) to include rare variants specifically associated with the EUR population [10]. The SNPs were mapped on different chromosomes to cover the human genome and avoid any linkage disequilibrium effects. The MAF of each SNP was retrieved from the Ensembl genome browser (v.111) (https://www.ensembl.org/index.html (accessed on 2 January 2025)). The SNPs to be included in the OA panel were also selected considering the availability of pre-designed TaqMan Assays on the manufacturer’s site (https://www.thermofisher.com (accessed on 2 January 2025)). A summary of the selected SNPs is provided in Figure 1 and Table S1.

### 2.3. DNA Purification and Quantification

The genomic DNA of the patients was extracted from peripheral blood or buccal swabs. Genomic DNA extraction was carried out utilizing the MagPurix^®^ 12A Instrument (Resnova, Rome, Italy) with the MagPurix^®^ Blood DNA Extraction Kit 200 for blood samples and the MagPurix^®^ Forensic DNA Extraction Kit for buccal swab samples.

A DS-11 FX spectrophotometer (DeNovix, Wilmington, DE, USA) was employed to assess the quality and concentration of each DNA sample. Concentration ranges of 10–150 ng/μL and A260/230 and A260/280 ratios between 1.7 and 1.9 were considered good parameters for DNA samples.

### 2.4. Whole-Exome Sequencing (WES)

Concerning WES analysis, the Illumina^®^ Next-Seq 550 system was utilized. In particular, 30–50 ng/μL of DNA was employed for library preparation by means of Illumina^®^ DNA Prep with Enrichment and Tagmentation kit according to the manufacturer’s instructions. The libraries obtained were sequenced at 2 × 100 bp, and the sequencing quality of the resulting data was expected to reach a quality score > 30 (Q30) for ~80% of the total called bases.

The sequencing reads were aligned to the reference genome hg19 using BaseSpace Variant Interpreter software (Illumina, v. 2.15.0.110). The resulting variants were visualized using Integrated Genome Viewer (v.2.7.2) and functionally annotated by means of BaseSpace Variant Interpreter (Illumina, v. 2.15.0.110), using GRCh37 as a genome build. Only the variants reporting a minimum coverage of 20xwere considered for subsequent analysis.

### 2.5. OpenArray™ Technology

OA technology is a rapid and accurate method for conducting high-throughput and large-scale systems biology studies with high specificity and sensitivity. In particular, the QuantStudio™ 12K Flex OA (( ThermoFisher Scientific, Waltham, MA, USA)) enables profiling, confirmation, and screening for various research applications, such as gene expression profiling and sample genotyping.

OA technology allows for the simultaneous genotyping of many samples using the QuantStudio™ 12K Flex system and the OpenArray™ Genotyping Analysis module. The QuantStudio™ 12K Flex system utilizes real-time PCR and TaqMan probes to target each SNP of the customized panel.

QuantStudio™ 12K Flex OA utilizes arrays consisting of 3072 through-holes. The OA system includes a specific plate (OA plate) that can analyze 48 to 192 samples in a single run. In fact, the OA block can accommodate up to four array plates per run [7].

A volume of 3 μL of extracted DNA (at a concentration of 10–50 ng/μL) and 3 μL of TaqMan OpenArray™ Genotyping Master Mix were manually loaded into 384-well plates following the manufacturer’s instructions. The mixture was then transferred to the TaqMan OpenArray™ plate using the QuantStudio™ 12K Flex OpenArray™ AccuFill System. The amplification process was performed using the QuantStudio™ 12K Flex Real-Time PCR System (Thermo Fisher Scientific, Waltham, MA, USA), and the results were analyzed using TaqMan Genotyper Software v1.4.0 (Thermo Fisher Scientific, Waltham, MA, USA). The software allowed evaluation of the functionality, amplification efficiency, and average call rate of each probe across experiments (National Research Council (US) Committee on DNA Technology in Forensic Science, 1992).

### 2.6. R Tool Description and Data Analysis

The concordance test was utilized to identify the percentage of matching SNPs between OA panel and WES data. To achieve this, we developed the Sample Tracking Tool, a custom R-based tool designed to facilitate sample matching and traceability. The tool performs data reading, matching, and merging of SNP data from different sources (OA and WES). The process includes aligning SNPs by genomic position (POS) and chromosome (CHR) to identify matches or mismatches between the output files obtained by the different platforms.

The tool filters data into two worksheets, MERGE and NO MERGE, and collects the results into a new Excel file. The MERGE worksheet contains data points with matched genotyping results between the OA and WES files, while the NO MERGE worksheet includes genotyping results from the OA panel that do not have a corresponding match in the VCF file. This is due to the nature of VCF files, which only report variants with alternative alleles, excluding homozygous wild-type (WT) calls. SNPs genotyped as homozygous WT in the OA file are included in the NO MERGE section and marked in green to indicate their expected absence in the VCF file. Discrepancies, such as mismatches in zygosity or unexpected calls, are flagged in red, highlighting SNPs requiring further verification.

**Figure 2:**
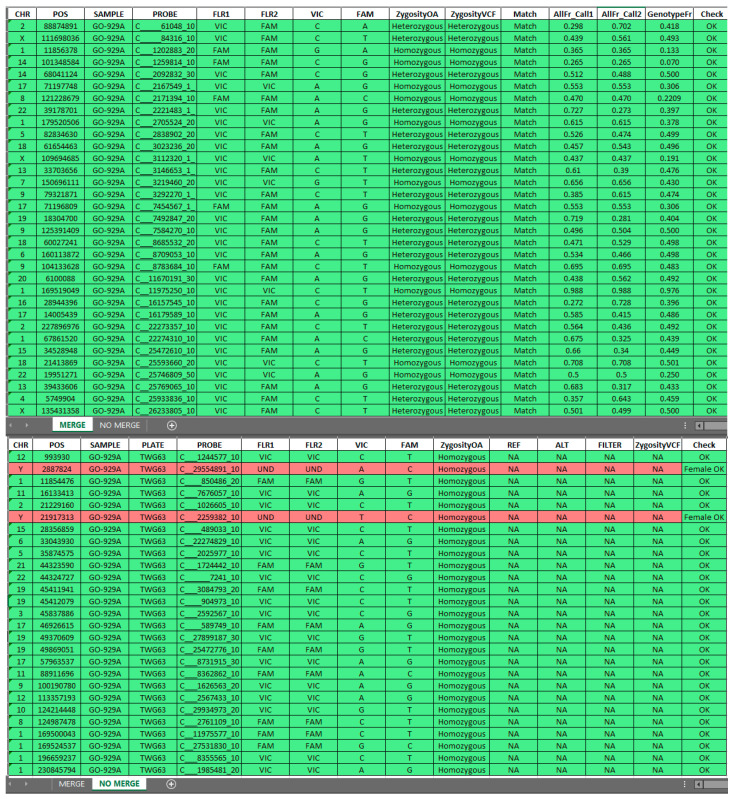
Output generated by the Sample Tracking Tool, divided into MERGE and NO MERGE sections. MERGE includes matched SNPs from the OA panel and WES data, while NO MERGE contains genotyping results without a corresponding match in the VCF file. SNPs genotyped as homozygous WT in OA data are included in NO MERGE (green rows) for clarity, while discrepancies are flagged in red for further review. This structure ensures comprehensive and accurate data interpretation.

Moreover, the tool streamlines data manipulation, integrates datasets, and calculates genotypic and allelic frequencies for matched SNPs based on Hardy–Weinberg equilibrium (HWE) principles. It also employs statistical methods to assess potential differences among SNP frequencies observed in the samples compared to the EUR general population. A practical illustration of the tool output is provided in Figure 2.

Moreover, the performance of the method was tested by estimating the random match probability (RMP). The random match probability (RMP) is a statistical measure used to evaluate the chance occurrence of an identical genetic profile. In the fields of forensic genetics and genetic identification, the RMP plays a pivotal role in assessing the discriminatory capacity of a genetic marker (or profile). The calculation of RMP employs genotypic frequencies and the principles of population genetics, particularly those defined by HWE. The RMP provides a statistical estimate of the probability that two individuals might share the same genotype purely by coincidence, thereby providing the discriminative power of the analyzed genetic markers. This comparison aids in assessing the suitability of a genetic marker for distinguishing between individuals in a population [11,12].

In addition to performing matching between OA and WES data, the R package *SNPassoc* (https://cran.r-project.org/package=SNPassoc (accessed on 2 January 2025)) was employed to evaluate potential differences in the frequency distribution of SNPs included in the OA panel compared to the EUR general population. Specifically, a chi-square test was applied to compare the observed allele frequencies in the study cohort with the expected frequencies based on the general European population. This step was essential to assess the suitability of the selected SNPs for individual identification and to ensure the absence of significant discrepancies in their allele frequencies. Statistical significance was considered with a threshold of *p* < 0.05, and results are summarized in Table S2

Further details on the use of the Sample Tracking Tool and the workflow can be found in File S1.

## 3. Results

The study was performed on 958 samples tested by WES and OA and subsequently analyzed using the Sample Tracking Tool for the matching process. In particular, the proposed protocol was based on the genotyping of 60 SNP panels, which were expected to enable the individual identification of specimens with a hypothetical RMP of 1.3398 × 10^−17^.

Genotyping analysis was initially tested on a cohort of 758 patients, revealing an average call rate of 94.4 ± 7.8% and a concordance rate of 97.19 ± 2.87% for the probes included in the panel (Table S3).

**Figure 3:**
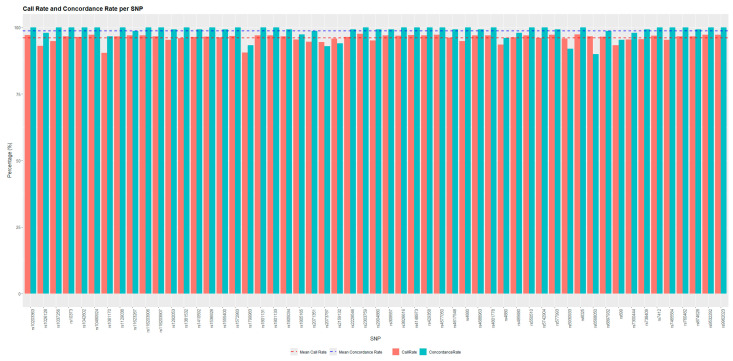
Comparison of call rate and concordance rate for each SNP. The x-axis lists the SNP IDs, while the y-axis represents the percentage values for call rate (red bars) and concordance rate (blue bars). The dashed line represents the average call rate and concordance obtained for the SNPs of interest.

The TaqMan Genotyper Software indicated that two probes (C__29554891_10, C___2259382_10) had a call rate <90% and did not pass the quality control. Additionally, the probe C___2171394_10 reduced amplification efficiency, while C___2592567_10 and C___2567433_10 displayed an apparently low efficiency in discriminating heterozygous genotypes. Although the TaqMan Genotyper Software highlighted homozygosity for these two probes, examination of the amplification plots revealed the presence of the two amplification curves expected for heterozygous samples. Consequently, these probes were excluded from subsequent analysis. The average call rate and concordance rate were then recalculated on the remaining 55 probes, resulting in 96.04 ± 1.5% and 97.53 ± 2.88%, respectively (Figure 3).

The application of the R package SNPassoc allowed for the comparison of allele frequencies for the SNPs included in the OA panel with those reported for the general European (EUR) population. The chi-square test applied to assess potential discrepancies did not report any statistically significant differences (*p* > 0.05) between the observed and expected allele frequencies. This finding confirms that the SNPs utilized in the OA panel are suitable for individual identification, as their frequency distribution aligns with the general EUR population. The detailed results of this analysis are provided in Table S2, where each SNP of interest is listed alongside its observed frequencies, expected frequencies, and corresponding *p*-values.

Successively, the OA and WES outputs were then run by the Sample Tracking Tool, which successfully performed the matching process and confirmed the sample’s identity. The protocol and Sample Tracking Tool were then validated on 100 additional samples. The calculation of the RMP of this cohort was conducted considering the 55 SNPs, revealing a value of 3.29 × 10^−15^, thereby confirming an appropriate discriminative power of these sets of SNPs for sample identification.

**Figure 4:**
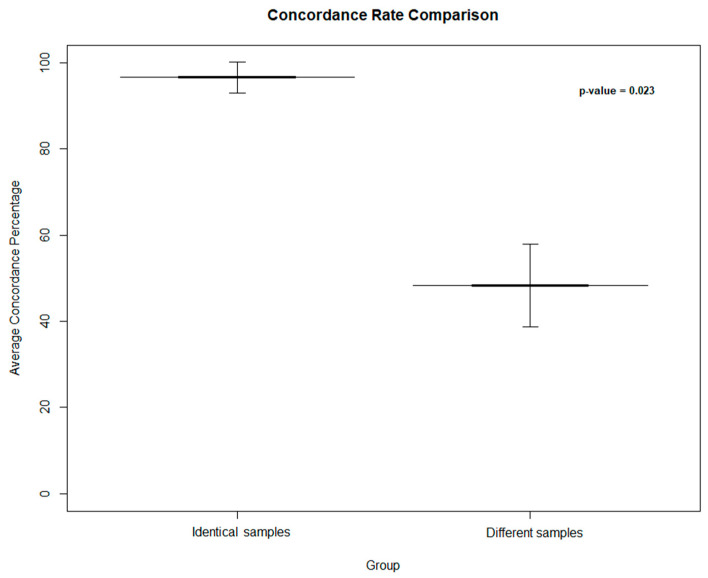
Average concordance rate between identical and different samples, presented as a bar plot with 95% confidence intervals. The ‘Identical’ group includes samples correctly matched, while the ‘Different’ group comprises samples matched with different identities. Shades of gray indicate the degree of concordance, with darker shades represent higher concordance. Error bars reflect the 95% confidence intervals around the mean values. The *p*-value represents the significance related to the concordance obtained by matching identical and different samples.

To further assess the performance of the protocol outlined in this study, a simulation was conducted on 100 additional samples by mixing the OA and WES data files related to this cohort and calculating the RMP and concordance rate. As a result, the average concordance rate was 48.31 ± 9.524% for different samples compared to that (96.63 ± 3.609%) resulting from identical samples. The difference between the concordance rates obtained from identical and different samples reported a significant *p*-value (*p* = 0.023) (Figure 4).

## 4. Discussion

**Figure 5:**
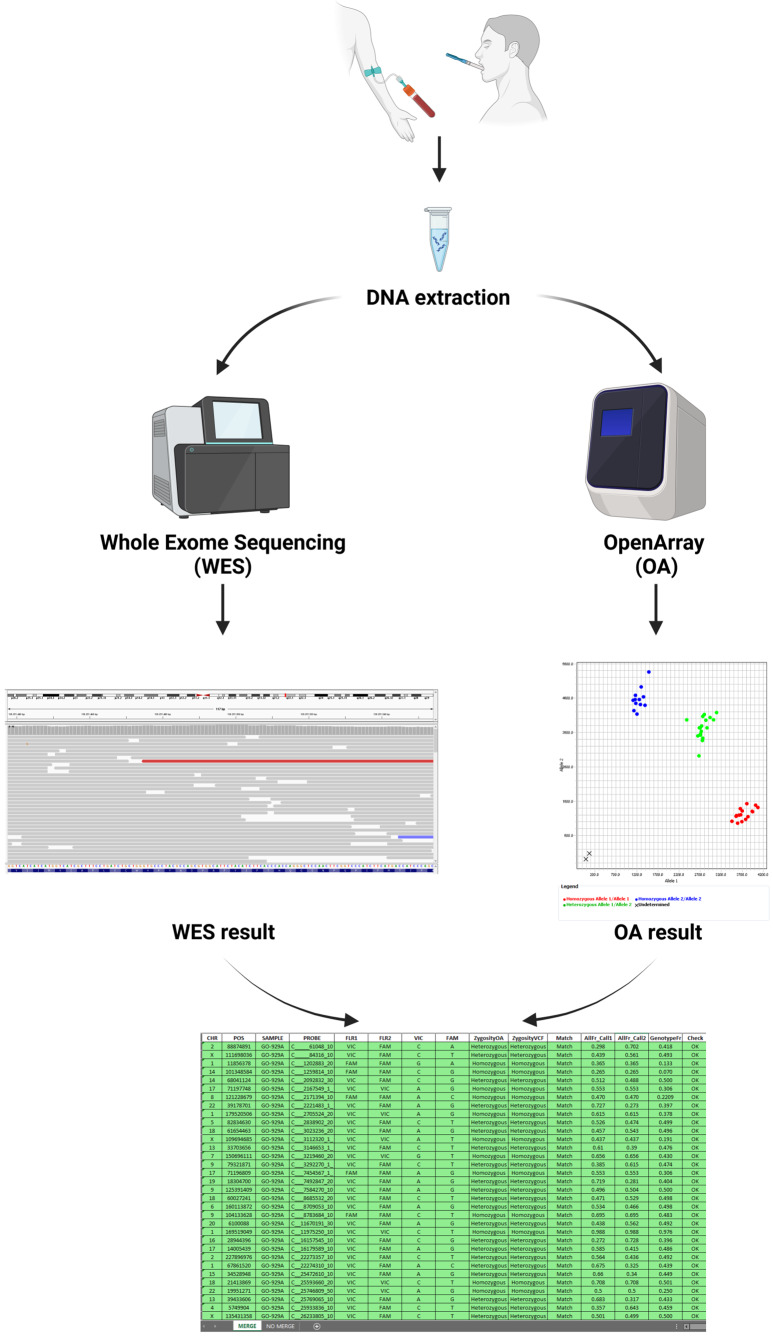
Illustration of the workflow for sample tracking. The DNA is extracted from blood samples or buccal swabs and subsequently analyzed using OpenArray^TM^ (OA) genotyping and next-generation sequencing (NGS). The resulting OA-generated data are compared to the NGS output data with the use of the Sample Tracking Tool in order to match the genotypes and confirm the sample identity. This figure has been created with Biorender.com (accessed on 2 January 2025).

In the last decades, the implementation of WES into clinical practice has significantly improved the molecular diagnosis of genetic disorders. The increasing demand for NGS to analyze a growing number of samples underscores the urgency of developing workflows that reduce the cost per test and turnaround time [13]. Centralizing genetic sequencing in specialized facilities is pivotal for reducing the costs associated with diagnostic testing, thereby enhancing the sustainability of genetic diagnostics [14]. This approach leverages economies of scale, streamlining processes and reducing redundancy in equipment and labor. In this context, the traceability of samples and quality control of data are of paramount importance to verify sample identity and avoid potential errors and mix-ups [15,16]. To meet regulatory requirements and ensure data monitoring, employing intrinsic sample and data identification methods provides a robust approach for processing many samples in a single lab setting. While good sample handling practices and increased laboratory automation help minimize the risk of errors, additional control steps are necessary to verify the identity of specimens. This study aims at developing a protocol (Figure 5) for tracking NGS-analyzed samples to prevent errors and mix-ups, ensuring the proper quality control, accuracy, and reliability in genetic testing procedures. The method was developed to select a set of SNPs as a source of genetic variations because of their extensive application in several fields, ranging from forensic purposes to population-wide studies [17]. In addition, a custom R tool (namely, Sample Tracking Tool) was designed for the systematic tracking and assessment of samples. The tool simplifies the process of sample matching and provides an accurate and automated analysis. The tool’s code is meticulously commented on and organized, demonstrating a commitment to clarity and ease of understanding. This automation not only saves time but also reduces the likelihood of manual errors, ensuring a reliable and smooth analytical process.

In this study, 60 SNPs were accurately selected based on their location in regions covered by the WES platform and with a MAF > 1% in the EUR general population. Subsequently, the RMP of the selected SNPs was calculated to estimate the expected discriminative power of the OA panel. As a result, the expected RMP was found to be sufficiently high (1.3398 × 10^−17^) and in line with other similar methods published in the literature [6,7,10]. Notably, Yousefi et al. [11] developed a 50 SNP panel exhibiting high discriminative power. The selection of SNPs was based on several criteria, including genotype identification rate, MAF, HWE, and linkage disequilibrium. This SNP panel yielded a probability of identity of 6.9 × 10^−20^ assuming no familial relationships and 1.2 × 10^−10^ when considering the presence of siblings.

Kidd et al. [18] described a set of SNPs that exhibited high levels of heterozygosity and limited frequency variation across different populations, which are highly desirable features for an individual identification panel. The authors selected markers considering their frequency among 40 populations. They chose 50 SNPs, resulting in an average match probability of approximately 10^−15^ with a narrower range overall.

In contrast to the OA panel presented in this study, the above-mentioned panels primarily comprise SNPs located within intragenic regions [7,11,17,18], making them unsuitable for applying them in the comparative analysis of genetic variants covered by WES platforms. This is the reason why the SNPs included in this study were primarily selected considering their localization in genomic regions covered by WES platforms.

After designing the OA panel, the method was tested on 758 samples. The analysis of TaqMan Genotyper Software indicated that five probes (C__29554891_10, C___2259382_10, C___2171394_10, C___2592567_10, C___2567433_10) had insufficient call rates and did not meet quality control standards. These SNPs were therefore not included in further analysis. In particular, the SNPs rs9786184 and rs2032652 showed a call rate < 90%, indicating potential challenges in genotyping reliability for these SNPs. Additionally, rs4870723 exhibited amplification issues, making it impossible to calculate the call and concordance rates. The rs2271615 and rs10774671 displayed low efficiency in discriminating heterozygous genotypes. Although the TaqMan Genotyper Software detected a signal expected for homozygous cases, careful examination of the amplification plots revealed the presence of two curves expected for the heterozygous samples [19]. Consequently, these two SNPs were excluded from further analysis to ensure the reliability of the genotyping data. The genotyping analysis yielded good results in the testing cohort, showing an average call rate of 94.4 ± 7.8%, indicating robust performance of the genotyping protocol. The high concordance rate of 97.19 ± 2.87% further emphasized the accuracy of the Sample Tracking Tool in confirming sample identities based on OA and WES outputs.

The protocol and Sample Tracking Tool were then validated on 100 additional samples. The RMP was calculated considering the 55 SNPs and revealed a value of 3.29 × 10^−15^, suggesting a high discriminatory power for sample identification. Indeed, this value means that the chance of observing a profile consisting of the genotypes referred to the 55 selected SNPs in the general population is less than 1 in 10 billion. To enhance the reliability of the genotyping approach, the R package *SNPassoc* was used to check the existence of potentially different frequency distributions of the SNP panel in our case cohort with respect to the EUR general population. Applying a chi-square test, the results (Table S3) revealed non-significant *p*-values (*p* > 0.05) for the SNPs of interest, confirming the suitability of the SNP panel for individual identification purposes. Moreover, the high concordance rate proved the reliability and robustness of the Sample Tracking Tool in providing accurate sample matching even for large-scale genomics studies and clinical applications.

The availability of the Sample Tracking Tool facilitated and accelerated sample matching processing. One of the advantages of the Sample Tracking Tool is to return, within the MERGE sheet, an RMP value of the single sample considering only the frequencies of the matching probes. This feature is used as a crucial step in assessing whether an individual sample falls within the expected RMP range for the panel. It aids in determining whether the hypothesis of the sample being the same or different can be accepted. This result highlights the tool’s ability not only to manage large-scale datasets but also to provide precise, reliable, and rapid discrimination at the individual sample level. The NO MERGE worksheet is not informative for checking sample identity, and it cannot be taken into consideration for the matching process.

Furthermore, the developed method was subjected to a simulation test performed on 100 additional samples in order to test different scenarios in which the samples matched accurately and did not. Accurately matched samples exhibited a remarkably high concordance rate (96.63 ± 3.609%), in contrast to mixed samples showing inappropriate concordance (48.31 ± 9.524%). This result further emphasizes the Sample Tracking Tool’s ability to distinguish between accurate and inaccurate matches, strengthening its reliability for individual identification.

The Sample Tracking Tool was also compared with several commercially available kits designed by different companies (such as NimaGen, Pxlence, Twist, Nijmegen, The Netherlands), showing main differences in terms of number of SNPs, labor intensity, and discrimination power [20]. The assay (“EasySeq™ Human DNA Sample Identification”) developed from NimaGen employs patented Reverse Complement PCR (RC-PCR) technology, which allows for simultaneous target amplification, sequencing adaptor addition, and sample indexing within a single closed-tube reaction. This kit exploits a panel of 40 targets, including 37 SNP (28 of them are shared with our protocol) and three gender markers and can be performed on extracted DNA or directly on blood samples. The Twist Human Sample ID Kit can also be applied starting from blood or purified genomic DNA and is based on the testing of 30 SNPs (9 of which are shared with our protocol) and 2 gender markers. It provides the reagents needed to prepare sample-tracking libraries using multiplex PCR amplification and ligation of adapters from the Twist Universal Adapter system. The Twist Universal Adapter System streamlines the library preparation process by combining multiple steps—adapter ligation, indexing, and amplification—into a cohesive workflow. However, this kit has been validated with other Twist NGS workflows (including Twist EF library prep and exome 2.0 panel), making its application restricted to the availability of these assays. The Human Sample ID Kit by Pxlence simplifies library preparation by utilizing a single PCR step starting from purified genomic DNA. This step simultaneously amplifies 50 targets, including 44 highly polymorphic SNPs and 6 gender markers, and adds specific barcodes to each sample. This streamlined process reduces the complexity and time required for library preparation.

Overall, these methods are more labor-intensive and expensive than our protocol, making them less feasible for diagnostic centers. Nimagen, Pxlence, and Twist kits are NGS-based technologies that take additional time and costs compared to our method because of the need for indices and pooling of samples into a single tube, increasing the risk of errors and operational complexity. In contrast to the above-mentioned methods based on targeted sequencing assays run on the same NGS platform, our protocol does not interfere with the workflow required for WES and allows proving sample identity by testing the same panel of SNPs analyzed by two independent technologies that ensure genetic data reliability in diagnostic laboratory settings. Moreover, our protocol can be applied in laboratories managing a small number of samples, whereas NGS-based commercial kits are cost-effective only in the presence of a huge number of samples.

Another critical aspect to consider in the comparison of DNA sample tracking methods is the software used for data analysis. The kits provided by Nimagen, Pxlence, and Twist require proprietary software that is tailored specifically to their platforms and the unique SNPs included in their panels. This software often come with additional costs and may lack flexibility for customization outside of its intended and validated design. In contrast, our matching tool solution is handcrafted, versatile, and allows for the modification of SNPs according to the specific needs of the laboratory or population of interest. Moreover, our tool was developed in R, an open-source programming language that is freely available and easily customizable for different applications. This flexibility and cost-effectiveness offer significant advantages to laboratories looking to tailor their DNA tracking systems to specific requirements without incurring additional expenses.

Finally, we compared the random match probability of all methods to evaluate the discriminatory power of the different solutions. The inclusion of 55 SNPs with a high call rate (≥90%) ensures robust discrimination power and a random match probability of 3.29 × 10^−15^, making our protocol highly reliable for preventing sample mix-ups and errors during NGS analysis. Pxlence also showed a very low RMP of 3.97 × 10^−18^, indicating excellent discriminatory capacity. In contrast, the Nimagen EasySeq™ Kit and Twist Sample ID Protocol exhibited higher RMP values of 1.01 × 10^−12^ and 4.41 × 10^−11^, respectively.

By comparing our protocol with existing commercial solutions, we highlighted its potential to provide a cost-effective, high-throughput, and reliable method for SNP-based sample tracking. Nevertheless, it presents some limitations related to the difficulty of distinguishing between samples from monozygotic twins, detecting maternal contamination, and resolving samples from closely related family members. In these challenging scenarios, further research studies are warranted to avoid sample mix-up and errors in these cases as well.

In conclusion, the results discussed in the present study further support the utility of the developed protocol for efficiently tracking samples and rapidly identifying any errors or mix-ups during the analytical processing. In addition, this method represents a valuable example for promoting laboratory centralization and minimizing the risks related to different laboratory procedures and the management of a high number of samples.

## Data Availability

The data presented in this study are openly available in GitHub at https://github.com/GiuliaC1995/Sample-Tracking-Tool (accessed on 2 January 2025).

## Supplementary Materials

The following supporting information can be downloaded at https://www.mdpi.com/article/10.3390/diagnostics15020149/s1: Table S1. List of single-nucleotide polymorphisms (SNPs) selected for designing the OA panel; Table S2. Observed and expected allelic frequencies for 55 SNPs in the European population; Table S3. Results related to the SNP panel include details on the call rate and concordance rate between OA and WES platforms; File S1. Sample Tracking Tool workflow.
