# Supplementary material for: Sample Tracking Tool: A Comprehensive Approach Based on OpenArray Technology and R Scripting for Genomic Sample Monitoring
Source: Diagnostics (Basel). 2025 Jan 10;15(2):149. doi: 10.3390/diagnostics15020149 (PMC11763353; doi:10.3390/diagnostics15020149)
Supplement: Supplementary file 1 [file diagnostics-15-00149-s001.zip › Table S1_round1.pdf]

Table S1. List of Single nucleotide polymorphisms (SNPs) selected for designing the OA panel.

SNP	MAF EUR	Position (GRCh38)	Gene	TaqMan AssayID
rs1801131	31%	1:11794419	MTHFR	C____850486_20
rs1801133	36%	1:11796321	MTHFR	C___1202883_20
rs118203907	0%	1:169530805	F5	C__11975577_10
rs6025	1%	1:169549811	F5	C__11975250_10
rs118203906	0%	1:169555299	F5	C__27531830_10
rs1410592	38%	1:179551371	NPHS2	C___2705524_20
rs1061170	36%	1:196690107	CFH	C___8355565_10
rs699	41%	1:230710048	AGT	C___1985481_20
rs2229546	33%	1:67395837	IL12RB2	C__22274310_10
rs5742904	0%	2:21006288	APOB	C___1026605_10
rs10203363	44%	2:227032260	COL4A4	C__22273357_10
rs1805165	30%	2:888575373	EIF2AK3	C_____61048_10
rs2271615	16%	3:45796394	SLC6A20	C___2592567_10
rs4688963	36%	4:5748177	EVC	C__25933836_10
rs6897932	27%	5:35874473	IL7R	C___2025977_10
rs309557	47%	5:83538811	VCAN	C___2838902_20
rs4880	47%	6:159692840	SOD2	C___8709053_10
rs2071351	19%	6:33076153	HLA-DPB1	C__22274829_10
rs1799983	34%	7:150999023	NOS3	C___3219460_20
rs4870723	47%	8:120216440	COL14A1	C___2171394_10
rs7465584	49%	8:123975238	FER1L6	C___2761109_10
rs1572983	31%	9:101371346	BAAT	C___8783684_10
rs1536928	50%	9:122629130	OR1B1	C___7584270_10
rs577993	38%	9:76706955	PRUNE2	C___3292270_1_
rs1381532	49%	9:97428498	TDRD7	C___1626563_20
rs10490924	19%	10:122454932	ARMS2	C__29934973_20
rs4617548	49%	11:16111867	SOX6	C___7676057_10
rs1042602	37%	11:89178528	TYR	C___8362862_10
rs10774671	35%	12:112919388	OAS1	C___2567433_10
rs7300444	44%	12:884764	WNK1	C___1244577_10
rs495680	39%	13:33129519	STARD13	C___3146653_1_
rs9532292	32%	13:38859469	FREM2	C__25769065_10
rs11623267	27%	14:100882247	RTL1	C___1259814_10
rs1555400	49%	14:67574407	GPHN	C___2092832_30
rs1129038	36%	15:28111713	HERC2	C____489033_10
rs4577050	34%	15:34236747	SLC12A6	C__25472610_10
rs2904880	27%	16:28933075	CD19	C__16157545_10
rs2159132	41%	17:14102122	COX10	C__16179589_10
rs550510	17%	17:48849253	CALCOCO2	C____589749_10
rs1292053	42%	17:59886176	TUBD1	C___8731915_30
rs1026128	45%	17:73200670	COG1	C___7454567_1_
rs1037256	45%	17:73201609	COG1	C___2167549_1_
rs9962023	29%	18:23833905	LAMA3	C__25593660_20
rs1805034	47%	18:62360008	TNFRSF11A	C___8685532_20

rs3826616	46%	18:63987229	SERPINB8	C___3023236_20
rs874628	28%	19:18193890	MPV17L2	C___7492847_20
rs429358	16%	19:44908684	APOE	C___3084793_20
rs7412	6%	19:44908822	APOE	C____904973_10
rs4801778	19%	19:48867352	PLEKHA4	C__27899187_30
rs2303759	24%	19:49365794	DKKL1	C__25472776_10
rs10373	44%	20:6119441	FERMT1	C__11670191_30
rs4148973	41%	21:42903480	NDUFV3	C___1724442_10
rs4680	50%	22:19963748	COMT	C__25746809_50
rs760482	27%	22:38782696	DNAL4	C___2221483_1_
rs738409	23%	22:43928847	PNPLA3	C______7241_10
rs2073787	44%	X :110451457	RGAG1	C___3112320_1_
rs6568050	44%	X :112454808	ZCCHC16	C_____84316_10
rs5930933	50%	X :136349199	ADGRG4	C__26233805_10
rs2032652	3%	Y:19755427	-	C___2259382_10
rs9786184	47%	Y:3019783	LINC00278	C__29554891_10
