# Supplementary material for: Sample Tracking Tool: A Comprehensive Approach Based on OpenArray Technology and R Scripting for Genomic Sample Monitoring
Source: Diagnostics (Basel). 2025 Jan 10;15(2):149. doi: 10.3390/diagnostics15020149 (PMC11763353; doi:10.3390/diagnostics15020149)
Supplement: Supplementary file 1 [file diagnostics-15-00149-s001.zip › Table S2_round2.pdf]

Table S2. Observed and Expected Allelic Frequencies for 55 SNPs in the European Population. This table presents the observed and expected allelic frequencies for 55 Single Nucleotide Polymorphisms (SNPs) in the European population. The chi-square test and corresponding p-values assess the statistical significance of the differences between the observed and expected frequencies.

	**Reference SNP Number**	**Frequencies for the European population**		**Frequencies for the study population**		***p_value***
		**Wild-type allele**	**Alternative allele**	**Wild-type allele**	**Alternative allele**	
1	rs1410592	G: 0.385	A: 0.615	G: 0.311	A: 0.689	0.879
2	rs2229546	C: 0.325	A: 0.675	C: 0.321	A: 0.679	0.895
3	rs10203363	C: 0.564	T: 0.436	C: 0.646	T: 0.354	0.592
4	rs4688963	T: 0.643	C: 0.357	T: 0.646	C: 0.354	0.592
5	rs309557	T: 0.474	C: 0.526	T: 0.533	C: 0.467	0.761
6	rs7465584	T: 0.513	C: 0.487	T: 0.566	C: 0.434	0.71
7	rs1381532	A: 0.488	G: 0.512	A: 0.503	G: 0.497	0.808
8	rs1536928	A: 0.496	G: 0.504	A: 0.526	G: 0.474	0.772
9	rs1572983	C: 0.305	T: 0.695	C: 0.295	T: 0.705	0.853
10	rs577993	C: 0.385	T: 0.615	C: 0.268	T: 0.732	0.81
11	rs4617548	A: 0.488	G: 0.512	A: 0.543	G: 0.457	0.745
12	rs7300444	C: 0.560	T: 0.439	C: 0.626	T: 0.374	0.62
13	rs495680	T: 0.390	C: 0.610	T: 0.394	C: 0.606	0.985
14	rs9532292	A: 0.683	G: 0.317	A: 0.649	G: 0.351	0.587
15	rs1555400	G: 0.488	C: 0.512	G: 0.411	C: 0.589	0.957
16	rs4577050	G: 0.340	A: 0.660	G: 0.351	A: 0.649	0.944
17	rs1026128	A: 0.447	G: 0.553	A: 0.440	G: 0.560	0.91
18	rs1037256	G: 0.447	A: 0.553	G: 0.457	A: 0.543	0.882
19	rs1292053	A: 0.583	G: 0.417	A: 0.477	G: 0.523	0.85
20	rs2159132	G: 0.415	A: 0.585	G: 0.394	A: 0.606	0.985
21	rs1805034	C: 0.471	T: 0.529	C: 0.411	T: 0.589	0.957
22	rs3826616	A: 0.457	G: 0.543	A: 0.457	G: 0.543	0.882
23	rs9962023	T: 0.292	C: 0.708	T: 0.255	C: 0.745	0.789
24	rs10373	A: 0.438	G: 0.562	A: 0.460	G: 0.540	0.878
25	rs4148973	T: 0.412	G: 0.588	T: 0.450	G: 0.550	0.894
26	rs760482	A: 0.727	G: 0.273	A: 0.712	G: 0.288	0.502
27	rs2073787	T: 0.563	A: 0.437	T: 0.623	A: 0.377	0.625
28	rs5930933	C: 0.501	T: 0.499	C: 0.523	T: 0.477	0.777
29	rs6568050	T: 0.561	C: 0.439	T: 0.520	C: 0.480	0.781

30	rs1061170	C: 0.362	T: 0.638	C: 0.368	T: 0.632	0.972
31	rs10490924	G: 0.805	T: 0.195	G: 0.801	T: 0.199	0.393
32	rs7412	C: 0.937	T: 0.063	C: 0.968	T: 0.132	0.286
33	rs429358	T: 0.845	C: 0.155	T: 0.934	C: 0.066	0.259
34	rs1801131	T: 0.687	G: 0.313	T: 0.712	G: 0.288	0.502
35	rs1801133	G: 0.635	A: 0.365	G: 0.50	A: 0.50	0.813
36	rs6025	T: 0.012	C: 0.988	T: 0.023	C: 0.977	0.457
37	rs550510	G: 0.827	A: 0.173	G: 0.838	A: 0.162	0.352
38	rs738409	C: 0.774	G: 0.226	C: 0.709	G: 0.291	0.506
39	rs4880	A: 0.534	G: 0.466	A: 0.546	G: 0.454	0.741
40	rs4801778	G: 0.808	T: 0.192	G: 0.811	T: 0.189	0.381
41	rs2071351	A: 0.814	G: 0.186	A: 0.805	G: 0.195	0.388
42	rs118203907	T: 1.000	C: 0.00003	T: 1.000	C: 0.000	0.206
43	rs118203906	C: 1.000	G: 0.0002	C: 1.000	G: 0.000	0.206
44	rs699	A: 0.588	G: 0.412	A: 0.523	G: 0.477	0.777
45	rs5742904	C: 0.999	T: 0.001	C: 0.987	T: 0.013	0.216
46	rs1042602	C: 0.628	A: 0.372	C: 0.596	A: 0.404	0.665
47	rs1129038	C: 0.365	T: 0.635	C: 0.599	T: 0.401	0.66
48	rs4680	G: 0.500	A: 0.500	G: 0.533	A: 0.467	0.761
49	rs11623267	C: 0.735	G: 0.265	C: 0.719	G: 0.281	0.492
50	rs6897932	C: 0.729	T: 0.271	C: 0.775	T: 0.225	0.423
51	rs874628	A: 0.719	G: 0.281	A: 0.639	G: 0.361	0.602
52	rs2303759	T: 0.760	G: 0.240	T: 0.652	G: 0.348	0.583
53	rs1799983	T: 0.344	G: 0.656	T: 0.414	G: 0.586	0.952
54	rs1805165	C: 0.298	A: 0.702	C: 0.228	A: 0.772	0.747
55	rs2904880	C: 0.272	G: 0.728	C: 0.232	G: 0.768	0.753
