# Supplementary material for: Sample Tracking Tool: A Comprehensive Approach Based on OpenArray Technology and R Scripting for Genomic Sample Monitoring
Source: Diagnostics (Basel). 2025 Jan 10;15(2):149. doi: 10.3390/diagnostics15020149 (PMC11763353; doi:10.3390/diagnostics15020149)
Supplement: Supplementary file 1 [file diagnostics-15-00149-s001.zip › Table S3_round2.pdf]

Table S3. Results related to the SNPs panel include details on the call rate and Concordance rate between OA and WES platforms. The rs9786184 and rs2032652 were found to have insufficient Call Rate (<90%). The SNP rs4870723 exhibited amplification issues (NA). making it impossible to calculate the call and concordance rates. Moreover. rs2271615 and rs10774671 displayed a low efficiency in discriminating heterozygous genotypes. Consequently. the SNPs showing amplification issues or insufficient call rate were subsequently excluded from further analysis.

Reference SNP Number	Assay Name	Call Rate	Concordance Rate
rs1410592	C___2705524_20	96.44%	99.33%
rs2229546	C__22274310_10	96.38%	99.33%
rs10203363	C__22273357_10	97.18%	100%
rs4688963	C__25933836_10	97.01%	99.33%
rs309557	C___2838902_20	96.98%	99.33%
rs4870723	C___2171394_10	NA	NA
rs7465584	C___2761109_10	95.30%	100%
rs1381532	C___1626563_20	95.89%	100%
rs1536928	C___7584270_10	96.58%	100%
rs1572983	C___8783684_10	96.71%	100%
rs577993	C___3292270_1_	97.20%	99.33%
rs4617548	C___7676057_10	96.12%	99.33%
rs7300444	C___1244577_10	95.40%	98%
rs495680	C___3146653_1_	96.27%	98%
rs9532292	C__25769065_10	97.20%	100%
rs1555400	C___2092832_30	96.32%	99.33%
rs4577050	C__25472610_10	97.21%	100%
rs1026128	C___7454567_1_	93.02%	98%
rs1037256	C___2167549_1_	94.91%	100%
rs1292053	C___8731915_30	95.34%	99.33%
rs2159132	C__16179589_10	95.85%	94%
rs1805034	C___8685532_20	96.63%	99.33%

rs3826616	C___3023236_20	96.88%	100%
rs9962023	C__25593660_20	97.23%	100%
rs10373	C__11670191_30	96.68%	100%
rs4148973	C___1724442_10	97.18%	100%
rs760482	C___2221483_1_	96.64%	100%
rs2073787	C___3112320_1_	94.45%	93.00%
rs5930933	C__26233805_10	95.85%	92.00%
rs6568050	C_____84316_10	96.63%	90.00%
rs9786184	C__29554891_10	50.45%	95.33%
rs2032652	C___2259382_10	58.48%	100%
rs1061170	C___8355565_10	90.48%	96.67%
rs10490924	C__29934973_20	97.21%	100%
rs7412	C____904973_10	96.83%	100%
rs2303759	C__25472776_10	97.58%	100%
rs1801131	C____850486_20	97.03%	100%
rs1801133	C___1202883_20	97.04%	100%
rs6025	C__11975250_10	97.35%	100%
rs550510	C____589749_10	97.01%	100%
rs738409	C______7241_10	95.55%	99.33%
rs4880	C___8709053_10	93.57%	96%
rs2271615	C___2592567_10	84.70%	93.33%
rs4801778	C__27899187_30	97.02%	100%
rs10774671	C___2567433_10	95.99%	82%
rs2071351	C__22274829_10	94.63%	98.67%
rs118203907	C__11975577_10	96.67%	100%
rs118203906	C__27531830_10	97.03%	100%
rs1042602	C___8362862_10	96.39%	100%
rs1129038	C____489033_10	96.65%	100%
rs4680	C__25746809_50	94.90%	100%
rs11623267	C___1259814_10	97.04%	98.67%

rs6897932	C___2025977_10	96.55%	98.67%
rs874628	C___7492847_20	96.63%	99.33%
rs1805165	C_____61048_10	95.49%	97.33%
rs2904880	C__16157545_10	95.08%	99.33%
rs699	C___1985481_20	93.28%	95.33%
rs5742904	C___1026605_10	96.00%	100%
rs429358	C___3084793_20	97.01%	100%
rs1799983	C___3219460_20	90.57%	93.33%
