# Supplementary material for: Sample Tracking Tool: A Comprehensive Approach Based on OpenArray Technology and R Scripting for Genomic Sample Monitoring
Source: Diagnostics (Basel). 2025 Jan 10;15(2):149. doi: 10.3390/diagnostics15020149 (PMC11763353; doi:10.3390/diagnostics15020149)
Supplement: Supplementary file 1 [file diagnostics-15-00149-s001.zip › File S1_round2.pdf]

Sample Tracking Tool Workflow

Input:

- VCF file containing exome data.
- Text file from the OA platform with genotyping results.
- Sample name to be matched.

Output:

Excel file with two worksheets:

- MERGE: Matching SNPs between OA and WES data.
- NO MERGE: SNPs with no corresponding match in the VCF file.

Steps:

1. Setup Environment:

   - Install and load required R packages: readxl (https://readxl.tidyverse.org/ (accessed on 7 January 2025)), dplyr (https://dplyr.tidyverse.org/ (accessed on 7 January 2025)), openxlsx (https://cran.r-project.org/web/packages/openxlsx/index.html (accessed on 7 January 2025)), vcfR (https://cran.r-project.org/web/packages/vcfR/vignettes/intro_to_vcfR.html (accessed on 7 January 2025)).
   - Set the working directory and specify paths for input files.

2. Prepare OA Data:

   - Read the OA text file and extract rows corresponding to the specified sample.
   - Create a structured dataframe (sample_df) with relevant columns (e.g., SAMPLE, PLATE, PROBE, etc.).

3. Integrate Panel Information:

   - Load panel data from an Excel file.
   - Merge OA data with panel information based on the PROBE column to add genomic details (e.g., CHR, POS).

4. Create Template File:

   - Save the merged OA data to an Excel file in the "Template_OA" folder.

5. Match with VCF Data:

   - Load the VCF file and extract relevant information (e.g., CHR, POS, Zygosity).

- Compare SNPs from the OA data (ZygosityOA) with those in the VCF file (ZygosityVCF) based on CHR and POS.

6. Categorize Results:

- MERGE: SNPs with matching zygosity and calls.
- NO MERGE: SNPs absent in the VCF.

7. Calculate Frequencies:

- Compute allele and genotype frequencies for matched SNPs using Hardy-Weinberg Equilibrium.
- Assign a frequency of 1 for SNPs with mismatches to exclude them from statistical analyses.

8. Generate Output:

Save results to an Excel file with two sheets:

- MERGE: Highlight matches in green and mismatches in red.
- NO MERGE: Mark homozygous WT SNPs in green and flag heterozygous or undefined SNPs in red.

9. Finalize Results:

- Add a "Check" column to indicate whether SNPs need further inspection (e.g., using IGV).
- Save the final file to the "Results" folder.
